# War and Infectious Diseases: Challenges of the Syrian Civil War

**DOI:** 10.1371/journal.ppat.1004438

**Published:** 2014-11-13

**Authors:** Sima L. Sharara, Souha S. Kanj

**Affiliations:** 1 Curriculum in Global Studies, University of North Carolina, Chapel Hill, Chapel Hill, North Carolina, United States of America; 2 Department of Internal Medicine, Division of Infectious Diseases, American University of Beirut Medical Center, Beirut, Lebanon; Duke University Medical Center, United States of America

## Overview

Syria's ongoing three-year civil war has displaced 6.5 million Syrians, left hundreds of thousands wounded or killed by violence, and created a vacuum in basic infrastructures that will reverberate throughout the region for years to come. Beyond such devastation, the civil war has introduced epidemics of infections that have spread through vulnerable populations in Syria and neighboring countries. In this article, we discuss the growing epidemics of poliomyelitis, measles, and cutaneous leishmaniasis in Syria and the region to examine the impact of conditions of war on the spread of infectious diseases in a public health emergency of global concern.

## Introduction

In March 2011, unrest from the Arab Spring found its way to Syria, interrupting over 40 years of political stability and igniting a civil war that continues to ravage the country with no end in sight [1]. Beyond direct casualties, war, particularly civil war, provides ideal conditions for outbreaks of infections, and Syria's ongoing three-year civil war has been no exception to this rule [2]. Measles, hepatitis A, leishmaniasis, poliomyelitis, meningitis, and scabies have spread through vulnerable populations in Syria and refugee camps in neighboring countries, creating a health crisis that will require immense resources to address (Table 1) [3]. Concurrently, the shattered medical infrastructure, the exodus of health care workers, and the deterioration of immunization programs have created a dangerous vacuum in essential health care provision [4]. In the context of Syria's devastated health care infrastructure, we will discuss the spread of infectious diseases, particularly poliomyelitis, measles, and cutaneous leishmaniasis, among Syrian civilians, refugees, and citizens of neighboring countries, to examine what is nothing short of a regional, and arguably global, public health emergency.

**Table 1:** Reported cases of communicable diseases per year between 2011 and 2014 in Syria, Lebanon, and Jordan.

	NUMBER OF COMMUNICABLE DISEASE CASES REPORTED PER YEAR													
	Syrian Arab Republic^a^				Lebanese Republic^c^				Syrian Refugees in Lebanon^c^		Hashemite Kingdom of Jordan^d^			
	2011	2012	2013	2014∧	2011	2012	2013	2014*	2013	2014*	2011	2012	2013	2014
**Poliomyelitis**	0	0	35^b^	1^b^	0	0	0	0	0	0	0	0	0	n/a
**Measles**	n/a	13	n/a	n/a	9	9	1760	219	232	92	30	24	205	n/a
**Cutaneous Leishmaniasis**	n/a	52,982	n/a	n/a	5	2	1033	381	998	364	136	103	146	n/a
**Hepatitis A**	n/a	2203	n/a	n/a	448	757	1551	738	220	127	418	509	1082	n/a
**Typhoid Fever**	n/a	1129	n/a	n/a	362	426	407	102	21	7	2	4	4	n/a

a Data obtained from the Syrian Ministry of Health website in the Quarterly Report of Communicable Diseases [30].

b Data obtained from the Global Polio Eradication Initiative website [16].

c Data obtained from the Epidemiologic Surveillance Department of the Lebanese Ministry of Public Health [26].

d Data obtained from the Communicable Diseases System on the Jordan Ministry of Health Website [25].

∧ 2014 Data last reported on 08/13/14 from the Global Polio Eradication Initiative website [16].

* 2014 Data last reported on 08/01/14 from the Epidemiologic Surveillance Department of the Lebanese Ministry of Public Health [26].

## The War on Health Care

Prior to the conflict, the health care system in Syria consisted of a government-run public system that provided mostly primary care services, with the private sector concentrated in urban areas providing the majority of advanced care services [1]. The past three decades were characterized by an improved capacity of the health system, as well as rapidly improving national health indicators such as a falling infant mortality rate and an increased child immunization rate. Yet the onset of the civil war led to the complete deterioration of the health infrastructure through the wide destruction of facilities, the shortage in health care personnel and medicines, and a lack of secure routes and transportation.

Rather than providing a safe place of care and refuge, the Syrian health care system has been integrated into the civil war battlefield. Both the regime's military forces and antigovernment armed groups have attacked and appropriated medical facilities as a tactic of warfare [5]. According to the World Health Organization (WHO), 40% of Syria's ambulances are destroyed and 57% of public hospitals are severely damaged, with 37% remaining out of service [6]. At least 160 doctors have been killed and hundreds jailed, leading to the emigration of an estimated 80,000 doctors [4]. The 90% of pharmaceutical needs that were locally produced prior to the conflict has now been reduced to only 10%, contributing to significant drug shortages in essential medications [1]. Such shortages, power outages, and the lack of security and mobility to seek care all contribute to the growing humanitarian crisis in Syria.

This health care crisis has extended beyond Syria's borders with one of the largest refugee crises since World War II [2]. Millions have entered the neighboring countries of Lebanon, Jordan, and Turkey in search of security. In Lebanon, over a million Syrian refugees currently represent a quarter of the country's population, residing among the local population in over 540 sites, as refugee camps are yet to be built [7]. In Jordan, there are 3,500 Syrian refugees crossing the border each day, with 20% residing in the Al Zaatari camp and the remaining 80% living in urban areas in the north of Jordan [8]–[10]. In both countries, Syrian refugees utilize local health care resources. This has significantly strained local health care systems with insurmountable demands ranging from continued chronic care to the management of spreading communicable diseases [11]. The underfunding of UNHCR (United Nations High Commissioner for Human Rights) and other humanitarian organizations has reduced the medical subsidies to refugees, leading many to forgo necessary yet unaffordable treatment [12]. In Syria and its neighboring countries, critical gaps in essential health care delivery continue to aggravate what has been described as the worst humanitarian crisis of the 21st century [6].

## War and Infectious Diseases

The conditions of war among civilian populations exacerbate risk factors for the spread of infections [13].

### Vaccine-preventable diseases: Poliomyelitis, measles, and an international wake-up call

The devastated health care infrastructure in Syria has hindered immunization programs, leaving millions of citizens vulnerable to vaccine-preventable diseases [14]. Vaccination coverage in Syria is estimated to have dropped from 91% in 2010 to as low as 45% in some regions by 2013, indicating rapid collapse of immunization systems in conditions of war [3]. Of the 1.8 million Syrian children born since the conflict, over 50% are unvaccinated [15]. Consequently, 36 cases of poliomyelitis have been officially reported in Syria after 15 years of eradication [16]. Although the opposition-held northeastern province of Deir Ez Zur has been the epicenter of the outbreak, cases have been encountered in rural areas of Damascus, Aleppo, and other regions [17].

The poliomyelitis virus lives in sewage, water, and contaminated food. In Syria, raw sewage is pumped directly into the Euphrates River, which provides drinking and washing water to villages and chlorination to decontaminate the water has been discontinued since 2012 [15]. The strain of poliomyelitis in Syria has been linked to the wild-type poliovirus 1 (WPV1) from Pakistan, which is suspected to have been introduced to Syria by Pakistani jihadist fighters [18], [19]. The same strain was detected in the sewage of Cairo in December 2012 and in sewage in Israel and the West Bank shortly thereafter, without any clinical cases thanks to high vaccination rates in these areas [20]. WPV1 can easily and undetectably spread, with only one in two hundred unvaccinated infected individuals developing acute flaccid paralysis [18]. WHO estimates that over 7,600 Syrians are currently infected, since poliomyelitis thrives in unsanitary, crowded conditions and among malnourished children [21].

In response to this outbreak, the biggest immunization campaign in the region's history led to the vaccination of over 2.7 million Syrian children and 23 million in neighboring countries [13]. The campaign employed the bivalent oral polio vaccine, which challenges a child's immune response with the two remaining types of virus and can be used in short intervals for acute outbreak responses [20], [22]. Nevertheless, the constantly migrating population, the lack of precise monitoring mechanisms, and besieged locations that remain out of reach to immunization threaten the success of this campaign. While cases are yet to be reported in Lebanon and Jordan, Iraq has confirmed the first case of poliomyelitis, after a 14-year hiatus, in northern Baghdad [23].

This poliomyelitis outbreak has helped focus international attention on the severity of the ongoing health crisis in Syria, as well as the importance of reinvigorating eradication campaigns in the endemic countries of Afghanistan, Pakistan, and Nigeria to prevent the recurrence of such outbreaks [17]. Polio in Syria has been declared a public health emergency that requires international efforts and solidarity to prevent a global epidemic.

The hampering of immunization efforts has also contributed to the spread of other vaccine-preventable diseases such as measles. Overcrowding, unsanitary conditions, and the efficient transmissibility of measles make the Syrian population highly susceptible to acquiring and spreading the infection. Measles has swept through Syria, including Aleppo and the northern regions, with over 7,000 confirmed cases [24]. This epidemic has not spared refugees in neighboring countries, even among highly vaccinated populations [14]. In Jordan, 24 cases of measles were reported in 2012, while over 200 cases were reported in 2013 [25]. In Lebanon, nine reported cases of measles in 2012 increased to 1,760 cases in 2013, only 13.2% of which were among Syrian refugees [26]. The growing rate of infection among Lebanese nationals reveals how conditions of unrest have exploited deficiencies in Lebanon's measles immunization coverage to contribute to a regional crisis. In response, the Lebanese Ministry of Public Health launched a national immunization campaign in April 2014 with over 4,200 trained volunteers administering vaccines for polio, measles, and rubella [27].

While ongoing vaccination campaigns have tried to address this epidemic, challenges continue to prevent adequate coverage. Unlike the poliomyelitis vaccine, which is relatively easy to transport and is administered orally, the measles vaccine must remain chilled and is administered by injection, posing a challenge for aid workers trying to reach vulnerable populations. Although immunization campaigns continue to deliver vaccines for measles and poliomyelitis to millions of adults and children in Syria and the surrounding region, the ongoing civil war restricts access to entire districts, threatens the lives of volunteers in the immunization campaign, and hinders efforts to quell the ongoing humanitarian crisis.

### Leishmaniasis and a region at risk

The risk factors for cutaneous leishmaniasis, including malnutrition, poor housing, population displacement, and poverty, are unfortunately all met in the case of the Syrian crisis, transforming the national epidemic of cutaneous leishmaniasis into a regional threat [28]. Cutaneous leishmaniasis has been endemic in parts of Syria, mainly Aleppo, for decades [29]. However, the Syrian conflict and vast population displacement has significantly increased the incidence of the vector-borne disease within Syria and spread this epidemic into neighboring countries. Reported cases of cutaneous leishmaniasis in Syria continue to rise, with the last official figure reporting 52,982 confirmed cases in 2012 [30].

**Figure 1:**
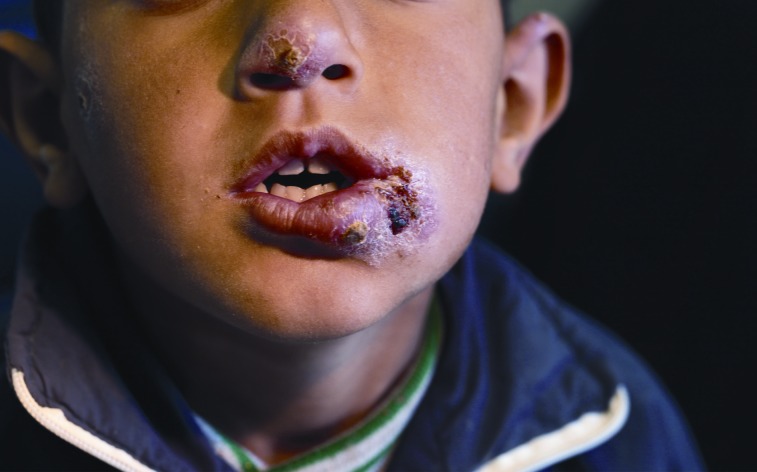
Syrian child from a Lebanon refugee camp, presenting multiple lesions from cutaneous leishmaniasis, courtesy of Dr. Ibrahim Khalifeh

Lebanon had no cases of cutaneous leishmaniasis before 2008 and sporadic cases in the following years. By 2013, 1,033 cases were confirmed, 96.6% (998) of which were among Syrian refugees [26]. In recently published data on 1,275 patients from 213 displaced Syrian families in Lebanon, the average age among infected individuals was 17 years, with many patients presenting with multiple disfiguring lesions (Figure 1). 77% of the patients manifested the disease after being in Lebanon for more than eight weeks, which is the known incubation period for cutaneous leishmaniasis, suggesting that the sand fly vector was transported to Lebanon with the incoming refugees [31]. Speciation by PCR showed that 85% of cases were caused by *Leishmania tropica*, with 15% of cases as *Leishmania major* [31].

The dense concentration of refugees, the similar environmental conditions, and the limited health care access in rural areas contribute to the higher burden of leishmaniasis in Lebanon. This epidemic has raised concerns that the sand fly vector may find a permanent habitat in Lebanon, particularly in rural areas with a high density of Syrian refugees such as the Bekaa valley, which holds 70% of all reported cases [32]. This outbreak has been the first of its kind in more than a decade and will continue to grow within the upcoming summer months if left unaddressed [32]. The Lebanese Ministry of Public Health (MOPH) and relevant governmental departments are planning a coordinated campaign to contain the spread of the infection, which includes spraying pesticides to kill the vector, providing free treatment and diagnosis for emerging cases and monitoring disease activity [32]. In April 2013, the Lebanese MOPH, in conjunction with WHO, organized workshops to train doctors across Lebanon to recognize the condition. In addition to the growing threat of leishmaniasis, experts have warned against the emergence of other vector-borne diseases such as dengue fever and malaria [33].

## A Civil War and a Global Threat

Without security, there can be no health. All efforts to quell the humanitarian crisis and to rebuild the broken health infrastructure in Syria will be largely futile as long as the civil war continues to rage on. The immediate end of war is inextricable from efforts to spare innocent lives and control this global threat of infectious diseases. Yet while the political borders of a conflict can be delineated, health care repercussions are uncontained by geopolitical borders. The spillover of refugees and communicable diseases into Lebanon, Jordan, and Iraq demonstrates the rippling consequences of the protracted Syrian conflict. In addition to the aforementioned infections, diseases such as typhoid fever, hepatitis A, meningitis, scabies, and lice continue to affect an increasingly vulnerable population. The international community has fallen short in its response to the crisis of infectious diseases in the Syrian conflict, and the consequences of this failure will continue to grow until there is a coordinated and exhaustive global effort.
